# Spasm of the Glottis in the New-Born

**Published:** 1910-01-08

**Authors:** J. H. Heaney


### The General Practitioner's Column.

[Contributions to this Column are invited, and, if accepted, will be paid for.]

### SPASM OF THE GLOTTIS IN THE NEW-BORN.

By J. H. HEANEY, M.B.

Attention is sometimes called by the nurse or mother to "choking" in infants a day or two old, but hitherto in my experience the condition has been trivial and temporary. In the two cases subjoined there was well-marked spasm of the glottis. Both proved fatal, and the symptoms appeared within two days of birth. Both children were girls, both full time and well nourished.

Case 1.—A first child. The membranes ruptured very early and complete dilatation of the os occupied about twenty hours. At the end of the twenty-first hour the child was delivered fairly easily with forceps. Respiration was induced artificially with little trouble. The breathing was accompanied with much rattling in the throat. This became much less marked during the next twenty-four hours, but never wholly disappeared. At this time the first well-marked spasm of the glottis occurred. The closure was complete and all the symptoms of suffocation were present for quite an appreciable time. Any attempt at swallowing brought on a fresh spasm. There was a complete absence of "crowing" or other sound. In the end the child died from exhaustion on the third day. The upper air passages only were examined post-mortem. There were no congenital deficiencies. Nothing whatever abnormal was found.

Case 2.—Also a first child, but born without assistance. The symptoms were precisely the same. Tracheotomy was performed at the hospital where I was acting as house-surgeon. Death on the fourth day. A post-mortem was not obtained.

Both these cases may be merely examples of laryngismus stridulus. The feeble respiratory apparatus at that early age could be made to account satisfactorily for the absence of "crowing." On the other hand, the train of symptoms may have been set up by the indrawing of mucus into the larynx, the child being too feeble thoroughly to clear the passage at any time afterwards. On this view various obvious methods of treatment short of operation were thought of and abandoned.

Tracheotomy was unsuccessful in Case 2, and does not seem to hold out much hope at such an early age. Intubation has recently been much discussed. Indeed, I have been led to publish these two cases chiefly in the hope of eliciting the opinion of laryngologists and others as to its probable efficacy under similar circumstances. In any case, operative treatment if adopted ought to be performed early. After two or three well-marked seizures, I believe it is useless to hope for recovery otherwise. The intervals of well-being suggest hope, but were in these two cases quite illusory.

